# CGM‐derived average glucose is more strongly associated with microvascular complications than HbA1c in type 1 diabetes

**DOI:** 10.1111/dom.70365

**Published:** 2025-12-09

**Authors:** Roland H. Stimson, Michael S. Crane, Anna R. Dover, Shareen Forbes, Rohana J. Wright, Marcus J. Lyall, Mark W. J. Strachan, Fraser W. Gibb

**Affiliations:** ^1^ Edinburgh Centre for Endocrinology & Diabetes NHS Lothian Edinburgh UK; ^2^ University/BHF Centre for Cardiovascular Science, Queen's Medical Research Institute University of Edinburgh Edinburgh UK; ^3^ Department of Clinical Biochemistry NHS Lothian Edinburgh UK

**Keywords:** continuous glucose monitoring (CGM), diabetic retinopathy, glycaemic control, type 1 diabetes

## Abstract

**Aims:**

To compare the associations of HbA1c and continuous glucose monitoring (CGM)‐derived average glucose with microvascular complications in adults with type 1 diabetes, and to assess the clinical utility and stability of metrics capturing glycation discordance.

**Materials and methods:**

Observational assessment of 9023 paired measurements of CGM data (14 days) and HbA1c in 2721 adults with type 1 diabetes. CGM metrics, HbA1c, and markers of discordance between HbA1c and CGM average glucose were associated with prevalent retinopathy (any and proliferative) and microalbuminuria.

**Results:**

HbA1c was higher than expected in older individuals (62 mmol/mol [54–71] age >45 vs. 61 mmol/mol [52–71], *p* = 0.004) and in women (62 mmol/mol [54–71] vs. 61 mmol/mol [53–71], *p* < 0.001) despite lower or similar average glucose levels. Fewer than one‐third of individuals remain within the same HbA1c—average glucose discordance category over time. HbA1c (*p* < 0.001), average glucose (*p* < 0.001), CV glucose (*p* < 0.001), and socioeconomic deprivation (*p* = 0.003) were all independently associated with retinopathy risk (with similar results for proliferative retinopathy). Higher glycation was associated with a lower likelihood of prevalent retinopathy (*p* < 0.001).

**Conclusions:**

CGM‐derived average glucose appears superior to HbA1c as a marker of prevalent microvascular complications. These data challenge the high‐glycator hypothesis and also suggest glucose variability may be an independent risk marker for microvascular disease.

## 1 INTRODUCTION

Since the Diabetes Control and Complications Trial (DCCT),^1^ glycated haemoglobin (HbA1c) has been the gold standard measure of glycaemic control and a primary target for therapy in type 1 diabetes. HbA1c is a robust predictor of long‐term risk for microvascular complications, including retinopathy, nephropathy, and neuropathy. However, it is a surrogate marker—representing the average glucose over the preceding 2–3 months—and may not capture the full complexity of glycaemic control, such as acute glucose fluctuations, postprandial spikes, and hypoglycaemic events.^2^, ^3^ Moreover, it can be influenced by non‐glycaemic factors such as red cell lifespan,^4^ iron status,^5^ and demographic variables including age,^6^ sex,^6^ and ethnicity.^7^ These sources of biological variation mean that, for individuals with identical mean glucose levels, HbA1c can differ significantly.

Continuous glucose monitoring (CGM) has transformed diabetes care by providing detailed data on glucose levels in real time. In addition to estimating average glucose, CGM‐derived metrics allow for a multidimensional assessment of glycaemic control. These metrics are increasingly used to guide therapy and are incorporated into consensus treatment targets.^8^ Glucose management indicator (GMI) is a mathematical manipulation of sensor average glucose and presents a value analogous to HbA1c.^9^ The value of GMI as an accurate reflection of HbA1c has been questioned.^10^, ^11^

Several approaches have been developed to quantify the discrepancy between HbA1c and CGM‐derived average glucose, including the haemoglobin glycation index (HGI)^12^ and glycation ratio (GR).^13^ These metrics aim to identify individuals in whom HbA1c may not accurately reflect glycaemic exposure—so‐called “high” or “low glycators.” If these metrics are consistent over time and independently associated with complication risk, they might offer additional value in personalising diabetes care. However, their clinical utility remains uncertain, and it is unclear whether they reflect a stable biological trait or are simply artefacts related to how GMI has been derived.

To date, few large‐scale studies have directly compared HbA1c and CGM‐derived metrics and their associations with established diabetes complications. Furthermore, it is not well understood whether metrics of glycation discordance and glycaemic variability add value to risk prediction models.

In this study, we sought to compare the relative associations of HbA1c and CGM‐derived average glucose with prevalent microvascular complications. We also explored whether metrics of glycation discordance and glucose variability were associated with complication risk. By doing so, we aimed to clarify whether HbA1c or CGM metrics are more clinically informative in assessing complication risk, and to what extent discordance metrics truly reflect a meaningful biological phenomenon.

## 2 METHODS

This was a retrospective, cross‐sectional assessment of adults with type 1 diabetes attending Edinburgh Centre for Endocrinology & Diabetes clinics (University hospital clinics serving approximately 5000 adults with type 1 diabetes). This project did not involve deviation from usual clinical care or access to data beyond our usual clinic IT systems, and ethical approval was not required.

The main cohort included 9023 paired CGM and HbA1c episodes in a total of 2721 individuals with type 1 diabetes (aged ≥18 and >6 months diabetes duration). The median number of paired data episodes was 3 (IQR 1–5) and data were collected between September 2018 and January 2024. CGM data relates to a 2‐week period of Freestyle Libre data obtained in the calendar month corresponding to the clinic HbA1c measurement. Paired data were only included in individuals with ≥70% CGM data available. The final paired data for each individual were used to assess the predictive performance of average glucose versus HbA1c with respect to proliferative retinopathy, any retinopathy, and persistent microalbuminuria (three or more elevated results). A cohort was established including only those with at least 3 paired CGM‐HbA1c measurements (*n* = 1404) to establish models for predictors of complications using the mean value of all measurements obtained for that individual.

Three methods were used to assess discordance between lab‐measured HbA1c and CGM‐derived metrics.

1. Haemoglobin glycation index (HGI) is defined as HbA1c minus GMI and a higher value indicates a higher HbA1c than would be predicted based on sensor glucose (so‐called high glycation).^12^
2. Glycation ratio (GR) is defined as GMI divided by HbA1c and a lower value with this metric indicates a higher HbA1c than would be predicted based on sensor glucose alone. Others have used a threshold of <0.9 to indicate high glycation status.^13^
3. Z‐score difference (zHGI): To allow direct comparison of HbA1c and GMI, both variables were standardised by conversion to z‐scores. For each metric, the z‐score was calculated as (value − cohort mean)/cohort SD. The discordance metric (zHGI) was defined as the difference between the HbA1c z‐score and the GMI z‐score, such that positive values indicate higher HbA1c than expected based on sensor‐derived glucose exposure.

Clinical and demographic data were obtained from SCI‐Diabetes (the Scottish national diabetes register), and CGM data were obtained from the LibreView portal. CGM metrics were assessed in line with the international consensus document on clinical CGM use.^8^ Scottish index of multiple deprivation (SIMD) is an area‐based measure of relative deprivation and is reported as rank (out of 6976‐1 is most deprived) and quintile (quintile 1 is most deprived). HbA1c was measured by cation‐exchange HPLC using either Menarini HA‐8180 or Tosoh G11 analysers according to standard laboratory protocol.

Data are presented as median (IQR) except glucose <3.0 mM (%) which is presented as mean (SD). Unpaired data were compared with Wilcoxon rank‐sum test. Categorical data were compared by *χ* ^2^ test, or McNemar's test when comparing paired data. Correlations were assessed by use of Spearman's correlation coefficient. Fisher r‐to‐z transformation was used to compare correlation coefficients. Associations between glycaemic metrics and prevalent microvascular complications (any retinopathy, proliferative retinopathy, and persistent microalbuminuria) were evaluated using multivariable logistic regression. Models were constructed using the mean HbA1c or mean CGM‐derived average glucose from all available paired measurements for each individual. Continuous predictors were modelled per unit increase. *P* values <0.05 were considered statistically significant. Statistical analyses were performed using R Studio (version 2023.12.1).

## 3 RESULTS

The total cohort included 2721 adults with type 1 diabetes. Median age was 45 years (IQR 32–57) and 53% were male. Median HbA1c was 61 (53–71). Twenty‐eight percent of were CSII users and median BMI was 26.9 kg/m^2^ (23.7–31.1). Full cohort characteristics are presented in Table S1, Supporting Information.

### 3.1 Assessment of glycation status

**FIGURE 1:**
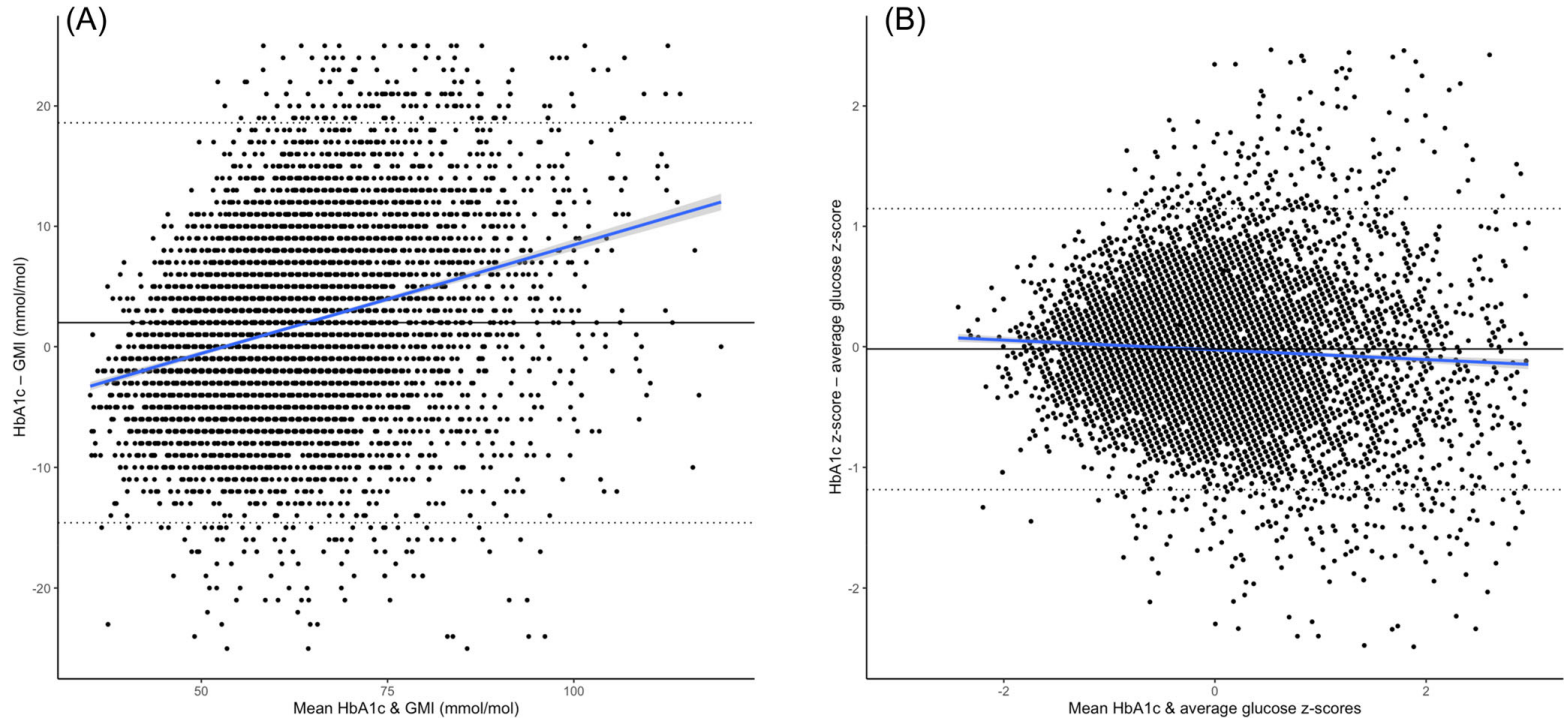
Bland–Altman plots for HbA1c–GMI discordance metrics. (A) Haemoglobin glycation index (HGI) across the range of GMI values. (B) Standardised z‐score difference (zHGI) showing the relationship between HbA1c and average glucose z‐scores. These plots highlight the patterns and directionality of discordance across glycaemic exposure levels.

Both HGI (R 0.552, *p* < 0.001) and GR (−0.542, *p* < 0.001) were strongly correlated with HbA1c. Neither was strongly correlated with GMI (R 0.035, *p* < 0.001 and R −0.017, *p* = 0.108, respectively). HGI values are presented on the y‐axis of the Bland–Altman plot (Figure 1A) and show that GMI is higher than HbA1c at the lower end of the range and lower than HbA1c at the higher end of the range (R 0.337, *p* < 0.001).

The equivalent plot where average glucose and HbA1c were normalized to z‐scores (y‐axis: zHGI) shows a clinically irrelevant (and opposite) correlation (R −0.055, *p* < 0.001) (Figure 1B). HbA1c is positively correlated with zHGI, but the relationship is weaker than with HGI (R 0.213, *p* < 0.001 and *p* comparing correlation coefficients <0.001). zHGI and GMI were significantly negatively correlated (R −0.325, *p* < 0.001 and *p* comparing HGI and zHGI correlation coefficients <0.001).

**FIGURE 2:**
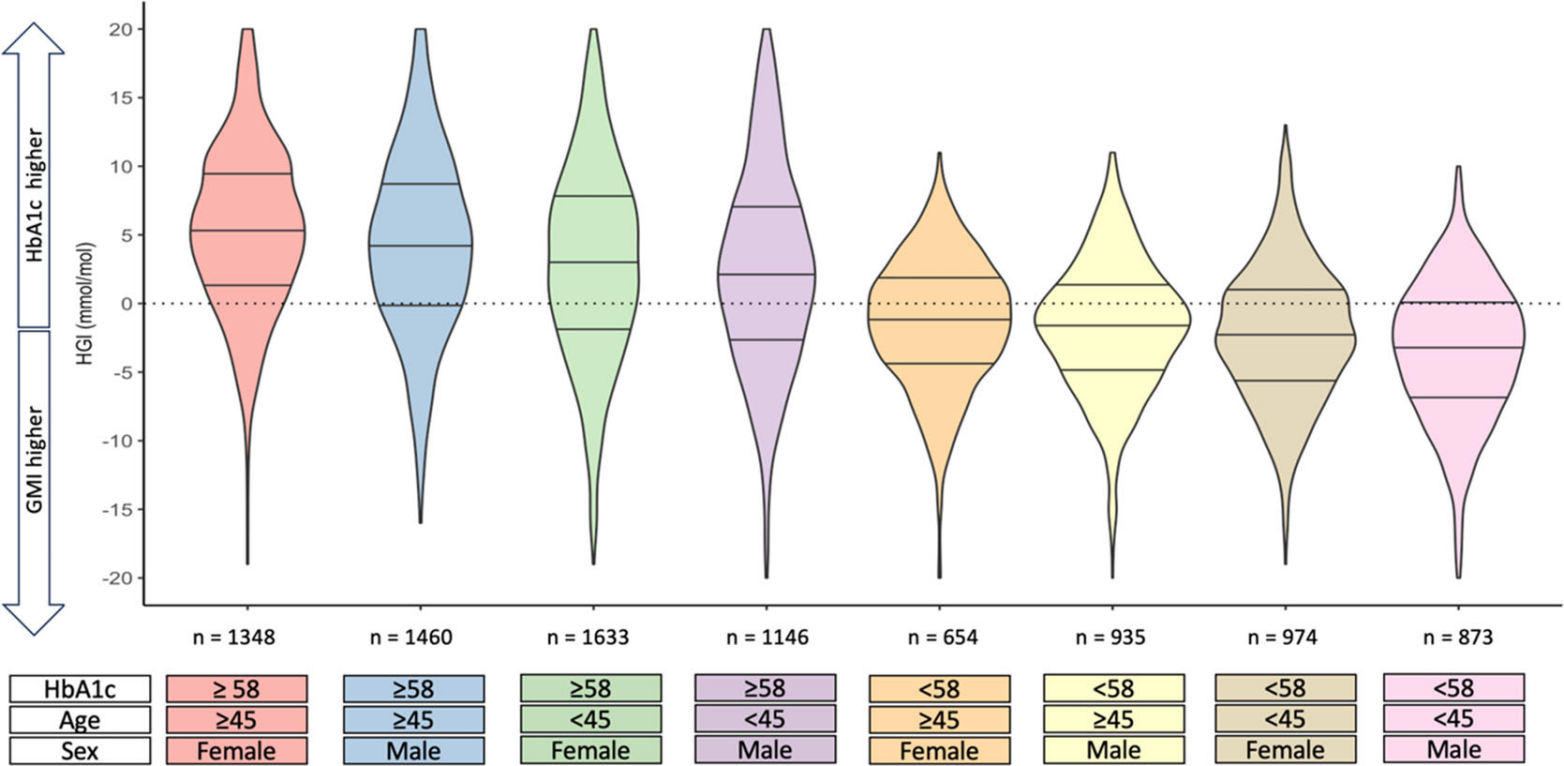
Impact of age, sex, and glycaemia on discordance between HbA1c and average glucose. HGI stratified by age group, sex, and HbA1c level. *p* < 0.001 for trend. *p* < 0.05 for all pairwise comparisons except 3rd vs. 4th category and 5th vs. 6th category, after adjustment for multiple comparisons. Horizontal lines in violin plots indicate median and interquartile range.

Whichever metric of discordance is used, there were significant relationships with age consistent with higher HbA1c in relation to sensor average glucose (HGI R 0.153, *p* < 0.001; GR −0.158, *p* < 0.001; zHGI R 0.173, *p* < 0.001). In individuals over the age of 45 years, HbA1c is higher than in younger individuals (62 mmol/mol [54–71] vs. 61 mmol/mol [52–71], *p* = 0.004) despite lower average glucose (9.8 mM [8.5–11.4] vs. 10.1 [8.6–11.9], *p* < 0.001). Similarly, women were more likely to have higher HbA1c (62 mmol/mol [54–71] vs. 61 mmol/mol [53–71], *p* < 0.001) despite no significant difference in average glucose (10.0 mM [8.6–11.6] vs. 9.9 mM [8.5–11.6], *p* = 0.418). This is reflected in significant differences in HGI (2 mmol/mol [−3 to 7] vs. 1 mmol/mol [−4 to 6], *p* < 0.001), GR (0.97 [0.90–1.05] vs. 0.99 [0.92–1.06], *p* < 0.001), and zHGI (0.01 [−0.32 to 0.33] vs. −0.06 [−0.38 to 0.26], *p* < 0.001). Considered together, the influence of HbA1c, sex, and age has a significant impact on discordance between HbA1c and CGM derived estimated HbA1c (Figure 2). The median HGI difference between women, aged ≥45 years, with HbA1c ≥58 mmol/mol and men, aged <45 years, with HbA1c <58 mmol/mol was 9 mmol/mol (*p* < 0.001).

**FIGURE 3:**
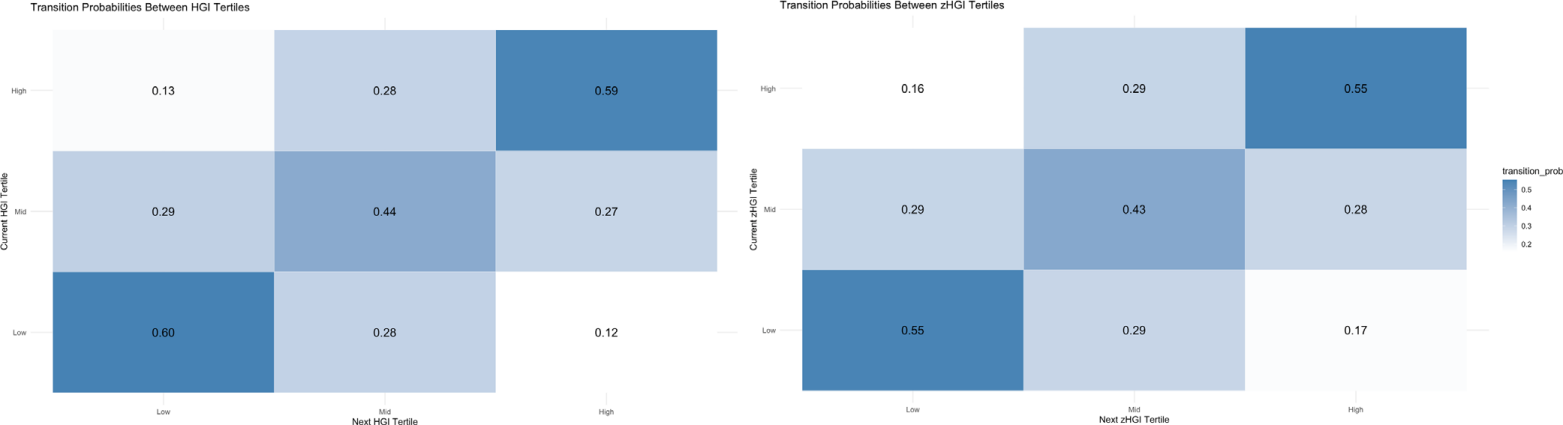
Transition probabilities across HGI categories over time. Each panel shows a heatmap of the likelihood of remaining in or transitioning between glycation index (HGI) categories from one timepoint to the next, among individuals with ≥3 measurements. Left panel: Transitions between raw HGI tertiles. Right panel: Transitions between standardised HGI (zHGI) tertiles. Rows indicate the starting tertile, columns the subsequent tertile. Cell values represent transition probabilities, with higher values along the diagonal reflecting greater category stability.

In individuals with three or more paired data points (*n* = 1404), the transition probabilities between one timepoint and the next for HGI and zHGI are presented in Figure 3. Over repeated measurement, only 22%, 18%, and 30% consistently remained within the same tertile for HGI, zHGI, and GR, respectively.

### 3.2 Associations with microvascular complications

In those with at least three paired CGM and HbA1c measurements (*n* = 1404), comparison of glucose metrics (mean of all measurements) and microvascular complications was assessed (Table 1).

**TABLE 1:** Differences in CGM, HbA1c, and discordance metrics according to microvascular complication status.

	Any retinopathy			Proliferative retinopathy			Microalbuminuria		
	Present (*n* = 776)	Absent (*n* = 547)	*p*‐value	Present (*n* = 204)	Absent (*n* = 1200)	*p*‐value	Present (*n* = 478)	Absent (*n* = 893)	*p*‐value
HbA1c (mmol/mol)	62 (55–71)	60 (53–69)	0.002	65 (59–77)	61 (54–69)	<0.001	64 (57–72)	60 (54–69)	<0.001
GMI (mmol/mol)	61 (55–68)	59 (53–65)	<0.001	64 (57–71)	59 (54–66)	<0.001	61 (55–68)	59 (53–66)	<0.001
Average glucose (mM)	10.2 (9.0–11.6)	9.8 (8.6–11.2)	<0.001	10.8 (9.5–12.4)	9.9 (8.7–11.2)	<0.001	10.3 (9.0–11.8)	9.9 (8.7–11.3)	<0.001
TAR (%)	47 (36–60)	43 (28–57)	<0.001	54 (40–65)	45 (31–57)	<0.001	48 (36–61)	45 (31–58)	<0.001
TIR (%)	49 (38–60)	53 (40–68)	<0.001	43 (33–56)	52 (41–65)	<0.001	49 (37–59)	52 (40–65)	<0.001
TBR (%)	3 (1–5)	2 (1–3)	<0.001	2 (1–5)	2 (1–5)	0.386	2 (1–5)	2 (1–5)	0.335
Time below 3.0 mM (%)	0.6 (1.4)	0.5 (1.2)	0.101	0.6 (1.4)	0.6 (1.4)	0.531	0.6 (1.3)	0.5 (1.3)	0.022
Time above 13.9 mM (%)	18 (9–29)	13 (5–24)	<0.001	22 (13–36)	15 (7–26)	<0.001	18 (10–30)	15 (7–26)	<0.001
CV (%)	38.0 (33.9–41.8)	35.5 (31.4–39.2)	<0.001	38.5 (33.6–42.5)	36.5 (32.5–40.6)	<0.001	37.8 (33.5–41.7)	36.5 (32.4–40.5)	<0.001
SD glucose (mM)	3.9 (3.3–4.5)	3.5 (2.9–4.1)	<0.001	4.2 (3.6–5.0)	3.6 (3.1–4.3)	<0.001	3.9 (3.3–4.5)	3.6 (3.3–4.3)	<0.001
Sensor active (%)	93 (88–96)	93 (88–97)	0.756	92 (86–96)	93 (88–96)	0.006	92 (87–96)	93 (89–97)	0.035
HGI (mmol/mol)	1.3 (−2.2 to 5.4)	1.5 (−1.7 to 5.3)	0.431	2.9 (−2.3 to 7.0)	1.3 (−1.8 to 5.3)	0.052	2.4 (−1.5 to 6.6)	1.0 (−2.0 to 4.7)	<0.001
GR	0.98 (0.92–1.04)	0.98 (0.92–1.03)	0.375	0.96 (0.91–1.04)	0.98 (0.92–1.04)	0.141	0.96 (0.91–1.02)	0.98 (0.93–1.04)	<0.001
Z‐score difference	−0.04 (−0.33 to 0.23)	0.03 (−0.27 to 0.30)	0.012	−0.03 (−0.39 to 0.29)	−0.01 (−0.30 to 0.27)	0.271	0.05 (−0.31 to 0.35)	−0.02 (−0.31 to 0.22)	0.030
Sex	Male 62% Female 56%	Male 38% Female 44%	0.046	Male 15% Female 14%	Male 85% Female 86%	0.577	Male 30% Female 40%	Male 70% Female 60%	<0.001
SIMD quintile (1 most deprived)	Quintile 1 72% Quintile 5 53%	Quintile 1 28% Quintile 5 47%	0.005	Quintile 1 21% Quintile 5 9.3%	Quintile 1 79% Quintile 5 91%	<0.001	Quintile 1 46% Quintile 5 34%	Quintile 1 54% Quintile 5 66%	0.057
Treatment type	CSII 61% MDI 58%	CSII 39% MDI 42%	0.334	CSII 12% MDI 16%	CSII 88% MDI 84%	0.128	CSII 32% MDI 37%	CSII 68% MDI 63%	0.072
Smoking status	Yes 61% No 58%	Yes 39% No 42%	0.600	Yes 18% No14%	Yes 82% No 86%	0.316	Yes 45% No 34%	Yes 55% No 66%	0.007
Diabetes duration at final HbA1c (years)	23.7 (16.8–32.2)	10.6 (4.4–21.1)	<0.001	27.7 (19.7–35.3)	17.6 (8.8–28.0)	<0.001	24.0 (15.3–33.8)	17.1 (8.2–26.9)	<0.001
Age at final HbA1c (years)	43.0 (32.7–53.3)	46.5 (31.6–58.1)	0.094	41.0 (33.2–54.2)	45.0 (31.6–55.5)	0.822	48.9 (33.9–57.2)	42.1 (31.3–54.0)	<0.001
BMI (kg/m^2^)	27.6 (24.4–32.3)	26.9 (23.9–30.2)	0.003	27.3 (23.5–32.7)	27.3 (24.2–31.1)	0.893	27.2 (24.0–32.0)	27.4 (24.2–31.2)	0.932

*Note*: Characteristics of individuals with and without any retinopathy, proliferative retinopathy, and microalbuminuria. Data are presented as median (IQR) except “Time below 3.0 mM (%)” which is presented as mean (SD) because the distribution is zero‐inflated, leading to median and IQR values of zero which do not reflect the underlying variability. Comparison of continuous variables between groups was by Wilcoxon rank‐sum test, and categorical variables were compared by χ^2^ test. Statistical significance was defined as *p* < 0.05.

In a logistic regression model, using the average of multiple measurements of CGM and HbA1c data, the significant predictors of any retinopathy were diabetes duration, CV glucose, socioeconomic deprivation, and either average glucose or HbA1c. In addition, zHGI (as a marker of discordance between HbA1c and CGM) was a significant predictor when average glucose was used, but more so with HbA1c (Table 2(A)). A similar pattern was observed in the logistic regression model predicting the presence of proliferative retinopathy. zHGI did not contribute to the model using average glucose but was a significant predictor in the model using HbA1c (Table 2(B)). Finally, the logistic regression model for microalbuminuria was similar, but socioeconomic deprivation was not a significant predictor whereas female sex was. zHGI was not significantly associated with microalbuminuria, whether HbA1c or average glucose was used as the glucose exposure metric (Table 2(C)). Notably, the direction of association of z‐score HbA1c minus z‐score glucose was in keeping with lower microvascular risk in so‐called “high glycators” with respect to all three outcomes assessed.

**TABLE 2:** Logistic regression models for associations with microvascular complications using HbA1c or average glucose.

(A) Any retinopathy						
	Model with HbA1c			Model with average glucose		
	HR	95% CI	*p*‐value	HR	95% CI	*p*‐value
HbA1c (mmol/mol)	1.02	1.01–1.03	<0.001	NA	NA	NA
Average glucose (mM)	NA	NA	NA	1.15	1.08–1.22	<0.001
Diabetes duration at final HbA1c (years)	1.07	1.06–1.09	<0.001	1.07	1.06–1.08	<0.001
CV (%)	1.06	1.03–1.08	<0.001	1.06	1.04–1.08	<0.001
SIMD 5 (least deprived)	0.74	0.56–0.98	0.033	0.74	0.57–0.92	0.033
zHGI	0.52	0.39–0.68	<0.001	0.70	0.53–0.92	0.012

(B) Proliferative retinopathy						
	Model with HbA1c			Model with average glucose		
	HR	95% CI	*p*‐value	HR	95% CI	*p*‐value
HbA1c (mmol/mol)	1.05	1.04–1.07	<0.01	NA	NA	NA
Average glucose (mM)	NA	NA	NA	1.37	1.26–1.48	<0.001
Diabetes duration at final HbA1c (years)	1.07	1.05–1.08	<0.001	1.07	1.05–1.08	<0.001
CV (%)	1.04	1.01–1.07	0.008	1.04	1.01–1.07	0.008
SIMD 5 (least deprived)	0.61	0.40–0.90	0.016	0.61	0.40–0.90	0.016
Gender	NA	NA	NA	NA	NA	NA
zHGI	0.49	0.34–0.69	<0.001	0.97	0.68–1.37	0.870

(C) Microalbuminuria						
	Model with HbA1c			Model with average glucose		
	HR	95% CI	*p*‐value	HR	95% CI	*p*‐value
HbA1c (mmol/mol)	1.03	1.02–1.04	<0.01	NA	NA	NA
Average glucose (mM)	NA	NA	NA	1.20	1.13–1.27	<0.001
Diabetes duration at final HbA1c (years)	1.04	1.03–1.05	<0.001	1.04	1.03–1.05	<0.001
CV (%)	1.03	1.01–1.05	0.007	1.03	1.01–1.05	0.007
Male sex	0.57	0.45–0.72	<0.001	0.57	0.45–0.72	<0.001
zHGI	0.84	0.65–1.09	0.191	1.25	0.97–1.62	0.089

*Note*: Hazard ratios (HR), 95% confidence intervals (CI), and *p*‐values for predictors of: (A) proliferative retinopathy, (B) any retinopathy, and (C) microalbuminuria. Models were constructed using mean HbA1c or mean average glucose across multiple paired measurements in 1404 individuals.

## 4 DISCUSSION

These data reflect important findings related to whether metrics of discordance between HbA1c and CGM‐derived average glucose reflect the underlying biology of an individual's haemoglobin glycation. These data also suggest that even a brief snapshot of glucose exposure (14 days of CGM data or a single HbA1c) was associated with underlying microvascular complications, with little difference between either modality. Analysing this in greater depth appears to challenge the “high glycator” hypothesis, and in fact suggests the opposite relationship may exist (i.e., so‐called “high glycation” is associated with lower prevalence of retinopathy). Furthermore, the fact that adjusting for glycation adds to the predictive power of HbA1c but not CGM‐derived average glucose suggests that adjusting HbA1c to better reflect average glucose may be a better marker of complications (at least for proliferative retinopathy). It was also notable that glucose variability (CV glucose) was an independent predictor of all complications.

We have shown that existing metrics used to describe discordance between HbA1c and CGM‐derived average glucose are rarely durable over repeated measurements within the same individual. In addition, age and sex are associated with discordance, meaning that a clinically relevant difference in HGI is present depending on the age, sex, and degree of hyperglycaemia. Normalising HbA1c and average glucose to z‐scores attenuates the HGI relationship with glycaemia (mean of HbA1c and average glucose) and, for that reason, it may be a superior metric of discordance. However, the fundamental unknown is whether sensor‐derived average glucose or HbA1c is the gold standard for glucose exposure. There are biological reasons why HbA1c may not accurately reflect glucose exposure in many individuals but HbA1c reporting is internationally standardised.^14^ On the other hand, the absence of standardisation for glucose measurement across different CGM systems^15^ raises concerns that assessment of discordance may vary markedly depending on the CGM system being used. This cohort confirms earlier findings showing that HbA1c is higher with increasing age and in women despite similar average glucose^6^; these associations may reflect differences in red cell turnover^4^ or iron status.^5^ Race is also known to be a predictor of discordance^7^ but our cohort is not racially diverse (largely white European) and did not permit further assessment of this association. Given the lack of durability of discordance measures within individuals (a small minority remained within the same tertile for either GR, HGI, or zHGI over repeated measures), it seems unlikely that these metrics are robust indicators of biological susceptibility to haemoglobin glycation.

Logistic regression analysis, particularly assessing proliferative retinopathy, provides the strongest hint that CGM‐derived average glucose has superior predictive power. Specifically, adjusting for HbA1c‐glucose discordance (zHGI) was not a significant predictor in the model using average glucose but was in the model using HbA1c. We suggest that this adjustment of the HbA1c‐based model, to better reflect the average glucose, hints at the superiority of average glucose over HbA1c. A similar trend was observed for all retinopathy but not with microalbuminuria. Differences with microalbuminuria may reflect a different risk profile for this complication, which was associated with age, female sex, and cigarette smoking in contrast to the assessed retinal complications. The association of microalbuminuria with female sex may be an artefact of selecting a threshold of 3.0 for elevated ACR, which is known to overestimate urinary albumin excretion in women.^16^

Another intriguing finding from this cohort was the association of all complications with glycaemic variability (CV glucose). It has been postulated that marked glycaemic excursions could drive complication risk by increasing oxidative stress^17^; however, attempts to demonstrate the impact of this in type 1 diabetes have typically been negative.^18^, ^19^ Although earlier studies were largely confined to assessments using 7‐point capillary blood glucose profiles, rather than CGM, the findings from our cohort are tentative and retrospective; it will be intriguing to establish, using prospective data, whether CGM‐derived glucose variability is a significant contributor to complication risk.

The association of retinal complications with greater socioeconomic deprivation is consistent with previously published evidence.^20^ Those with socioeconomic disadvantage may have had less access to preventative therapies or may have had greater exposure to hyperglycaemia in the long periods not captured within this data set.

Notwithstanding the retrospective nature of these data, the results from this cohort strongly suggest that the “high glycator” hypothesis is not a major contributor to the risk of complications in T1 diabetes. Whether or not currently available metrics of “glycation” are appropriate, the clear association in adjusted analysis suggested a relationship in the opposite direction (i.e., complication prevalence was lower in so‐called “high glycators”). We speculate that falsely reassuring HbA1c may result in therapeutic inertia and failure to appropriately intensify therapy. This is consistent with the findings of Shah et al.^12^ who found no association with high glycation and the development of retinopathy. GR has been shown to associate with skin advanced glycation end products (measured by skin autofluorescence) in people with type 1 diabetes.^13^ However, those with “high‐glycation” were significantly older (consistent with our cohort), and associations with skin autofluorescence did not persist after adjustment for age.

As alluded to, brief snapshots of glycaemic data are limited in their ability to predict outcomes in relation to years or decades of diabetes compared to prospective, cumulative data. However, the key aim of this investigation was not to develop predictive tools but to assess whether there were substantial differences in the ability of HbA1c or CGM‐derived metrics to predict prevalent complications. It could be argued that 2–3 months of CGM data would be more directly comparable to HbA1c; however, we were not able to obtain these data at scale. We have previously shown that a single episode of CGM average glucose data correlates as well as data captured over three consecutive months with respect to HbA1c.^6^ It is recognised that longer duration of CGM data may provide more robust data with respect to CV glucose and hypoglycaemia metrics.^21^ If anything, the shorter duration of CGM capture is likely to have underestimated the performance of CGM metrics compared to HbA1c and is unlikely to have introduced a type 1 error. In addition, 2‐week CGM data summaries are typically employed in clinical practice, and it is reassuring to observe that this brief duration of data collection performs as well (if not better) in predicting prevalent retinopathy. There is increasing evidence that different CGM systems are not adequately standardised^22^ and it is possible that results may be different when considering other sensors. However, it is potentially a strength of this study that all CGM data was consistently obtained from the same sensor system.

## 5 CONCLUSION

Our data suggest that CGM‐derived metrics are likely to be at least as reliable as HbA1c in helping direct therapy in type 1 diabetes to reduce complication risk. They also help inform clinicians of specific circumstances where HbA1c and average glucose are more likely to be discordant. These data also present tentative proof that glucose variability may be an independent risk factor for microvascular complications. It will be important to see if these observations are confirmed in long‐term prospective series.

## CONFLICT OF INTEREST STATEMENT

FWG has received personal fees from Abbott Diabetes Care, Dexcom and Roche. ARD has received personal fees from Abbott Diabetes Care.

## Supporting information


**Table S1.** Clinical and CGM characteristics of the total cohort. Data are presented as median (IQR) except “Time below 3.0 mM (%)” which is presented as mean (SD). HbA1c and CGM metrics refer to the last observation recorded.

## Data Availability

The data that support the findings of this study are available on request from the corresponding author. The data are not publicly available due to privacy or ethical restrictions.
