# Supplementary material for: CGM‐derived average glucose is more strongly associated with microvascular complications than HbA1c in type 1 diabetes
Source: Diabetes Obes Metab. 2025 Dec 9;28(3):1836–43. doi: 10.1111/dom.70365 (PMC12890757; doi:10.1111/dom.70365)
Supplement: Supplementary file 1 — Table S1. Clinical and CGM characteristics of the total cohort. Data are presented as median (IQR) except “Time below 3.0 mM (%)” which is presented as mean (SD). HbA1c and CGM metrics refer to the last observation recorded. [file DOM-28-1836-s001.docx]

**Supplementary table 1.** Clinical and CGM characteristics of the total cohort
Data are presented as median (IQR) except ‘Time below 3.0mM (%)’ which is presented as mean (SD). HbA1c and CGM metrics refer to the last observation recorded.

|  | Total cohort (n = 2721) |
| --- | --- |
| HbA1c (mmol/mol) | 61 (53 – 71) |
| GMI (mmol/mol) | 60 (53 – 67) |
| Average glucose (mM) | 10.0 (8.6 – 11.6) |
| TAR (%) | 46 (29 – 62) |
| TIR (%) | 51 (36 – 65) |
| TBR (%) | 2 (1 – 5) |
| Time below 3.0mM (%) | 0.6 (1.5) |
| Time above 13.9mM (%) | 15 (6 – 29) |
| CV (%) | 36.4 (31.9 – 41.4) |
| SD glucose (mM) | 3.7 (3.0 – 4.4) |
| Sensor active (%) | 94 (88 – 97) |
| HGI (mmol/mol) | 1 (-3 – 6) |
| GR | 0.98 (0.91 – 1.06) |
| zHGI | -0.02 (-0.35 – 0.30) |
| Sex | 53% male / 47% female |
| SIMD quintile | 10% most deprived  32% least deprived |
| CSII use | 28% |
| Current smoker | 12% |
| Diabetes duration at final HbA1c (years) | 19.8 (10.2 – 30.5) |
| Age at final HbA1c (years) | 45.0 (32.3 – 57.1) |
| BMI | 26.9 (23.7 – 31.1) |
